# Classification of salivary gland biopsies in Sjögren’s syndrome by a convolutional neural network using an auto-machine learning platform

**DOI:** 10.1186/s41927-024-00417-3

**Published:** 2024-11-06

**Authors:** Jorge Álvarez Troncoso, Elena Ruiz-Bravo, Clara Soto Abánades, Alexandre Dumusc, Álvaro López-Janeiro, Thomas Hügle

**Affiliations:** 1 Systemic Autoimmune Diseases Unit, Hospital Universitario La Paz, Madrid, Spain; 2 Pathology Department, Hospital Universitario La Paz, Madrid, Spain; 3 Department of Rheumatology, Lausanne University Hospital (CHUV), Lausanne, Switzerland; 4 University of Lausanne, Lausanne, Switzerland; 5 Pathology Department, Clínica Universidad de Navarra, Pamplona, Spain; 6 Servicio de Medicina Interna, Hospital Universitario La Paz, Paseo de la Castellana, 261, 28046 Madrid, España

**Keywords:** Artificial intelligence, Salivary gland biopsy, Sjögren, Sicca

## Abstract

**Background:**

The histopathological analysis of minor salivary gland biopsies, particularly through the quantification of the Focus Score (FS), is pivotal in the diagnostic workflow for Sjögren's Syndrome (SS). AI-based image recognition using deep learning models has demonstrated potential in enhancing diagnostic accuracy and efficiency in preclinical research.

**Objectives:**

The primary aim of this investigation was to utilize an auto-machine learning (autoML) platform for the automated segmentation and quantification of FS on histopathological slides, aiming to augment diagnostic precision and speed in SS.

**Methods:**

A cohort comprising 86 patients with sicca syndrome (37 diagnosed with SS based on the 2016 ACR/EULAR Classification Criteria and 49 non-SS) was selected for an in-depth histological examination. A repository of 172 slides (two per patient) was assembled, encompassing 74 slides meeting the classificatory thresholds for SS (FS ≥ 1, indicative of lymphocytic infiltration) and 98 slides showcasing normal salivary gland histology. The autoML platform utilized (Giotto, L2F, Lausanne Switzerland) employed a Convolutional Neural Network (CNN) architecture (ResNet-152) for the training and validation phases, using a dataset of 172 slides.

**Results:**

The developed model exhibited a reliability score of 0.88, proficiently distinguishing SS cases, with a sensitivity of 89.47% (95% CI: 66.86% to 98.70%) and a specificity of 88.24% (95% CI: 63.56% to 98.54%). The model found histological slides of suboptimal quality (e.g., those compromised during fixation or staining processes) to be the most challenging for accurate classification.

**Conclusion:**

AutoML platforms offer a rapid and flexible approach to developing machine learning models, even with smaller datasets, as demonstrated in this study for SS. These platforms hold significant potential for enhancing diagnostic precision and efficiency in both clinical and research settings. Multicentric studies with larger patient cohorts are essential for thorough evaluation and validation of this innovative diagnostic approach.

## Key messages

- Histopathological analysis of minor salivary gland biopsies, especially the quantification of the Focus Score (FS), is crucial for the diagnostic workflow in Sjögren's Syndrome (SS).

- AI-based image recognition and deep learning models have shown promise in enhancing diagnostic workflows and have potential applications in preclinical research

- This study utilizes an auto-machine learning (autoML) platform for automated delineation and quantification of FS on histopathological slides, aiming to improve diagnostic precision and speed in SS.

- Offers a potential solution to mitigate the challenge of interobserver variability in FS assessment, thus strengthening the diagnostic criteria and potentially improving diagnostic accuracy in SS.

- The flexibility and agility of autoML platforms like Giotto could expedite the diagnostic process, presenting a viable tool for clinicians and researchers, even with smaller datasets.

## Introduction

Sjögren's Syndrome (SS) is a chronic autoimmune disorder primarily characterized by exocrine gland dysfunction, yielding xerostomia and keratoconjunctivitis sicca. The etiology encompasses genetic predispositions, environmental, and hormonal factors, manifesting a global prevalence of 0.5–1%, with a female predilection [1].

Clinically, SS spans from isolated sicca symptoms to systemic involvement impacting the nervous system, kidneys, and lungs. A significant subset of patients exhibit extraglandular manifestations (EGMs), denoting systemic disease extension. EGMs include renal, pulmonary, muscular, and neurologic involvements, associated with elevated risks of severe complications like cardiovascular disease, vasculitis, and lymphomas, with a 6% lymphoma occurrence in primary SS patients showcasing systemic disease [1, 2].

EGMs in SS display extensive heterogeneity, with any organ or system susceptible to involvement, escalating the disease's complexity and potential severity [3]. Management of EGMs is organ-specific, tailored to the severity of manifestations, aimed at symptom alleviation, complication prevention or management, and quality of life enhancement [4].

Diagnostic endeavors in SS are challenged by diverse clinical presentations and the absence of a singular definitive diagnostic test, notably in seronegative patients lacking typical autoantibodies like anti-SSA/Ro [2]. This accentuates the necessity for thorough clinical evaluations and a multidisciplinary approach for efficacious management of both glandular and extraglandular manifestations, ameliorating the overall prognosis and quality of life for SS patients. The 2016 American College of Rheumatology/European League Against Rheumatism (ACR/EULAR) classification criteria have been instrumental in standardizing the diagnosis [5]. These criteria include a combination of clinical, serological, and histopathological features, with a focus on salivary gland biopsy as a key diagnostic tool. Histologically, SS is characterized by lymphocytic infiltration, acinar destruction, and fibrosis in the salivary glands. The focus score (FS), which quantifies the number of lymphocytic foci per 4 mm2 of glandular tissue, is a critical parameter for diagnosis. A FS greater than 1 is highly specific for SS [5]. FS correlates with higher humoral inflammation, antibody positivity, and lymphoma risk in SS patients [6].

The integration of Artificial Intelligence (AI) in healthcare, especially in pathology, has ushered in a transformative era, enhancing diagnostic accuracy and efficiency. With over 500 AI-based algorithms approved by the U.S. Food and Drug Administration, primarily for diagnostic support, AI's role is becoming pivotal. In the context of AI applications in histopathology, delineation typically involves segmenting specific regions of interest within an image, requiring manual annotations, while quantification involves measuring specific features, such as the number of lymphocytic foci, which can be achieved using threshold algorithms. Particularly in pathology, Convolutional Neural Networks (CNN) have shown promise in automating complex histological image interpretation, catering to the demands of modern pathology. The ability of AI to process large datasets, identify intricate patterns, and provide high-speed, accurate predictions underscores its value [7, 8].

Salivary gland biopsy interpretation in SS stands to benefit significantly from AI integration. AI image recognition can enhance precision and efficiency by identifying specific histopathological features [9] crucial for SS diagnosis and classification, such as focal lymphocytic sialadenitis, acinar atrophy, and fibrotic changes. By quantifying the FS, AI algorithms provide an objective measure, mitigating the challenges of interobserver variability, a notable issue in SS diagnosis. A deep learning algorithm has been presented that predicts the FS on histological slides of salivary gland biopsies obtained from 327 SS patients with an accuracy of 0.87 [10].

As a more recent development, automated AI platforms enable clinicians and scientists without coding experience to generate algorithms for image recognition purposes. Algorithms generated on autoML platforms can provide similar results to those created by data scientists [11, 12]. In one study, automated deep learning models for medical image classification were created by healthcare professionals without coding experience using Google AutoML and showed good performances, at least on internal datasets [13].

In conclusion, the amalgamation of AI, particularly algorithms like the Giotto model, with pathology practices, presents a promising avenue to surmount the diagnostic challenges posed by SS. Through accurate and objective analysis of histopathological features, AI algorithms not only streamline the diagnostic process but also contribute to better understanding and management of SS, heralding a significant stride towards precision medicine in systemic autoimmune diseases.

## Methods

### Clinical dataset and labeling

**Fig. 1:**
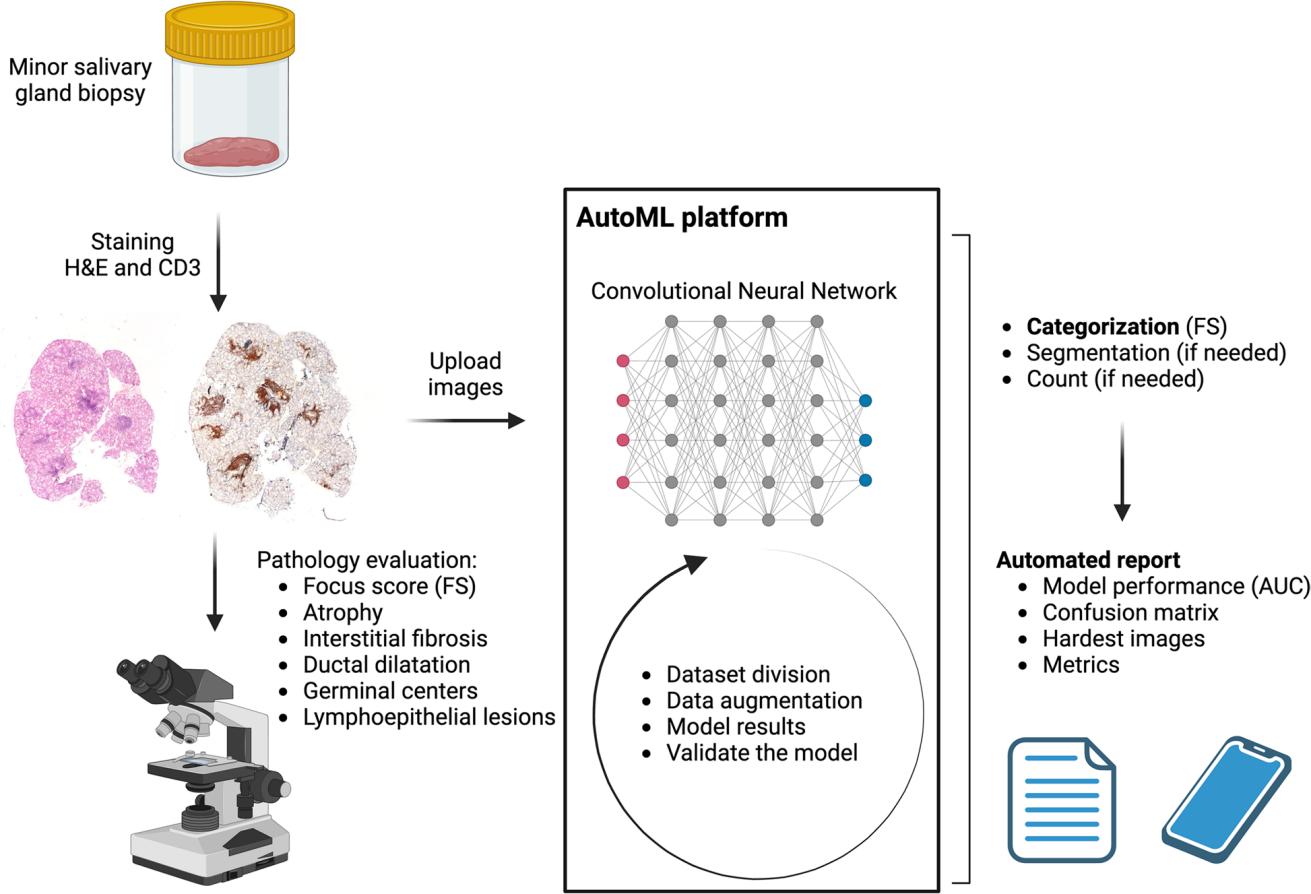
Giotto AI-platform training, validation and report

In this study, a cohort of 307 patients with sicca syndrome was evaluated using a multiparametric protocol that included Schirmer's test, Whole Unstimulated Salivary Flow (WUSF), immunological study, and salivary gland biopsy. Of these, 43% (132) were diagnosed with SS based on 2016 ACR/EULAR Classification Criteria [5]. Histological work up was performed by an expert pathologist (Fig. 1). Slides were labeled based on histopathological criteria. Each slide was labeled according to whether it represented SS (FS ≥ 1) or non-SS (normal histology). This labeling was based on expert pathologist evaluations to ensure accuracy. Exclusion criteria encompassed patients with coexisting conditions like sarcoidosis, IgG4-related disease, or lymphoma. Ethical approval was obtained from the Ethics Commission of Hospital Universitario La Paz (PI-5756), aligning with the stipulations of the Declaration of Helsinki.

### Hypothesis

The hypothesis posited that the CNN model would attain an Area Under the Curve (AUC) of 0.85 or higher in discerning one or more lymphocyte foci consistent with an SS infiltrate.

### Histology

**Fig. 2:**
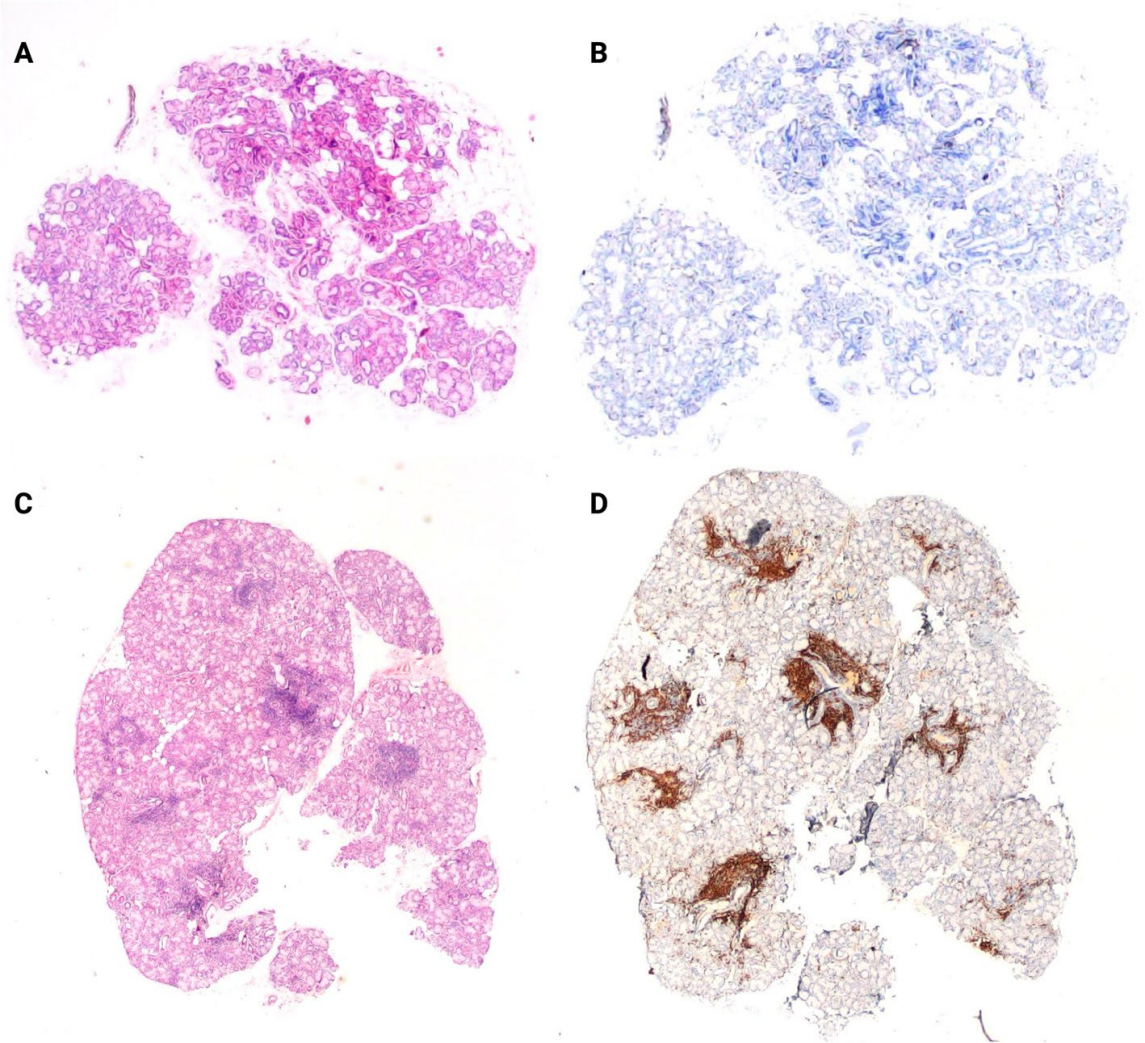
Non-Sjögren (**A**, **B**) and Sjögren (**C**, **D**) salivary gland histological samples stained with H&E (**A**, **C**) and CD3 (**C**, **D**)

Histological specimens from minor salivary glands of 86 selected patients (37 with SS and 49 with non-SS sicca syndrome) underwent staining with Hematoxylin–Eosin (H&E) and CD3 + , and were captured in panoramic imagery under standardized settings of brightness, contrast, with a magnification of 2x (Fig. 2). H&E staining provides essential details of tissue architecture and cellular morphology for diagnosing SS. CD3 staining labels T-lymphocytes, aiding in the precise quantification of lymphocytic infiltrates. Using different stainings helps train the model to recognize structures, not just colors and ensures comprehensive training data, enhancing the model's ability to generalize. The 2X magnification balances detail capture and computational load, ensuring efficient processing by the Convolutional Neural Network (CNN) architecture.

A compilation of 172 images (2 per patient) was created, which included 74 images indicative of SS (FS ≥ 1 indicating lymphocyte infiltrates) and 98 images representing normal salivary glands. Two slide sets from each group were treated with both H&E and CD3 staining, serving as the training material for the software, and yielded comparable outcomes from both sets. The images were subsequently partitioned into training (138 images, 80%) and validation datasets (34 images, 20%) for further analysis.

### AutoMachine Learning (autoML) platform

The Giotto algorithm follows a three-phase process: 1) Data collection and labeling: Slides were labeled based on histopathological criteria for SS (FS ≥ 1 for SS, < 1 for non-SS). 2) Data preprocessing and augmentation: This included techniques such as rotation, contrast adjustment, and zoom to enhance model training. 3) Model training utilizing CNNs: Specifically, a ResNet-152 architecture was employed.

Post-training, it quantitatively analyzes the FS and other histopathological features, enabling accurate and timely SS diagnosis, even in seronegative patients (Fig. 1).

Model performance was assessed using metrics such as accuracy, sensitivity, specificity, and the Area Under the Receiver Operating Characteristic Curve (AUROC). The dataset was divided into training and validation sets with an 80/20 split. The division ensured no overlap of patients between sets to avoid data leakage. Data augmentation was performed post-division to ensure robust training. Validation metrics were assessed at the end of each epoch to monitor model performance. A final independent test set is suggested for assessing generalization ability, though this was beyond the current study's scope.

Following an automated image preprocessing procedure augmented with data, a CNN architecture based on the ResNet-152 model was employed. The model, with a size of 178.43 MB, was trained over a span of 10 epochs.

The Giotto platform provides various metrics, including the loss evolution during model training for both training and validation datasets, information about the total number of samples, and the proportional division between training and validation sets. It also includes data on the dataset sizes in kilobytes (KB) and the class distribution across different datasets. Furthermore, a detailed tabulation is available, specifying all applied transformations, such as rotation, contrast adjustment, vertical flip, horizontal flip, random zoom, brightness, symmetric warp, and random crop.

For predictive and classification accuracy assessment, a confusion matrix was generated to evaluate the model's efficacy in accurately and inaccurately categorized images. With certain images, classification challenges were identified, with continuous monitoring of metrics on the validation dataset throughout the training regimen.

This study adheres to the TRIPOD (Transparent Reporting of a multivariable prediction model for Individual Prognosis Or Diagnosis) guidelines to ensure comprehensive reporting of the prediction model. Additionally, the CLAIM (Checklist for Artificial Intelligence in Medical Imaging) guidelines were followed to detail the development and validation of our AI-based image recognition model.

### Statistical analysis

Descriptive and inferential statistics were employed for data summarization and analysis. Categorical variables were tabulated as frequencies and percentages, while continuous variables were articulated as means and standard deviations (SD) for normally-distributed data, or medians and interquartile ranges (IQR) for skewed data distributions. The threshold for statistical significance was established at p-values less than 0.05. Analytical proceedings were conducted utilizing R-4.3.1 for Windows and Wizard Pro for Mac Version 2.0.12.

## Results

Table 1 presents demographic, clinical, immunological, and histological data between the SS and non-SS groups. Notable differences were observed in Anti-Ro positivity, Anti-La positivity, and Rheumatoid Factor positivity between the two groups (*p* < 0.001, *p* < 0.001, and *p* = 0.005, respectively). The Focus Score (FS) ≥ 1 was found to be significantly associated with SS (*p* < 0.001).

**Table 1:** Demographic, clinical, immunological and histological data of sicca syndrome patients

	**Sjögren (37)**	**Non-Sjögren (49)**	***p* value**
**Female Gender**	94.59%	87.76%	0.280
**Age ≥ 50 years**	67.57%	65.31%	0.826
**Associated systemic autoimmune diseases**	24.32%	24.49%	0.986
**Thyroid involvement**	43.24%	30.61%	0.227
**Neurological involvement**	13.51%	12.24%	0.862
**Pulmonary involvement**	5.41%	6.12%	0.888
**Renal involvement**	2.70%	0%	0.247
**Antinuclear Antibody (ANA) positivity**	70.27%	51.02%	0.072
**Anti-Ro/SSA positivity**	59.46%	2.04%	< 0.001
**Anti-La/SSB positivity**	29.73%	2.04%	< 0.001
**Rheumatoid factor positivity**	37.84%	12.24%	0.005
**Glandular atrophy**	75.00%	67.35%	0.444
**Interstitial fibrosis**	75.00%	73.47%	0.874
**Ductal dilatation**	66.67%	77.55%	0.264
**Germinal centers**	2.78%	0%	0.241
**Lymphoepithelial lesion**	13.89%	0%	0.007
**FS ≥ 1 (Focus Score ≥ 1)**	100%	0%	< 0.001
**Pathological schirmer**	75.68%	75.00%	0.943
**Pathological WUSF**	83.78%	68.09%	0.099

The algorithm with information on training and validation of the datasets is shown in Fig. 1. The training dataset comprised 138 images (83 Non-SS, 55 SS) with a total data size of 40.07 MB, while the validation dataset comprised 34 images (15 Non-SS, 19 SS) with a total data size of 10.02 MB (Table 2).

**Table 2:** Training and validation dataset info

Dataset	Total samples	Split (%)	Size
**Training dataset**	138 - 83 Non-Sjögren - 55 Sjögren	80%	40.07 MB
**Validation dataset**	34 - 15 Non-Sjögren - 19 Sjögren	20%	10.02 MB
**Total**	172 - 98 Non-Sjögren - 74 Sjögren	100%	50.09 MB

**Fig. 3:**
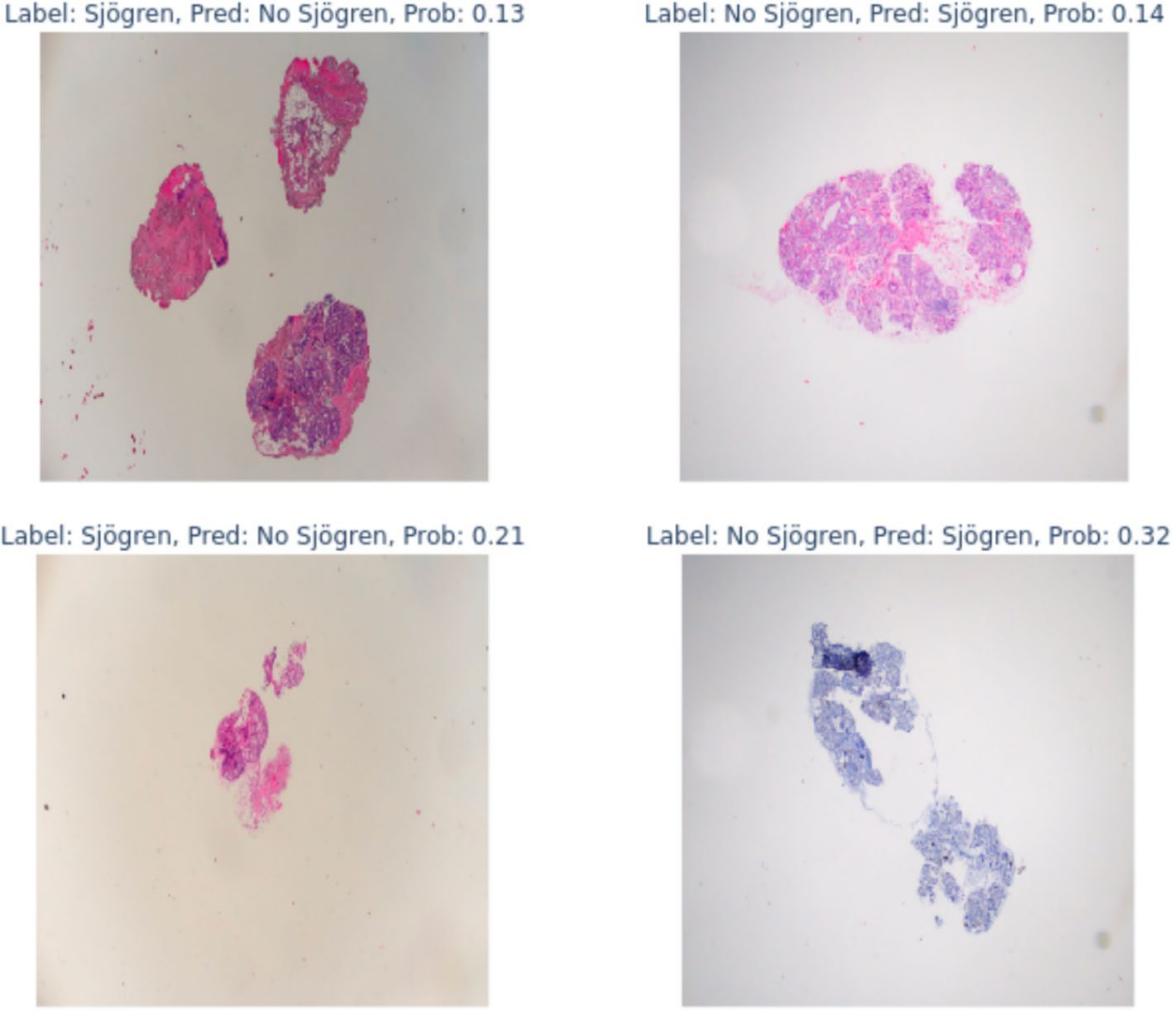
Hardest images for the model to classify correctly

Through the evaluation utilizing a confusion matrix, the model manifested a reliability of 88% (score 0.88), accurately discerning 17 out of 19 SS cases and 15 out of 17 non-SS cases. Furthermore, the system exhibited an adeptness at identifying more intricate images, subsequently ascertaining the likelihood of accurate classification as depicted in Fig. 3.

In the conducted evaluation, the diagnostic model demonstrated a sensitivity of 89.47% (95% Confidence Interval [CI]: 66.86% to 98.70%) and a specificity of 88.24% (95% CI: 63.56% to 98.54%). The Positive Likelihood Ratio was observed to be 7.61 (95% CI: 2.05 to 28.21), while the Negative Likelihood Ratio was ascertained at 0.12 (95% CI: 0.03 to 0.45). Within the studied population in Spain, the prevalence of the disease was documented at 0.50% [14]. The Positive Predictive Value was 3.68% (95% CI: 1.02% to 12.42%), whereas the Negative Predictive Value was notably high, at 99.94% (95% CI: 99.78% to 99.98%). The overall accuracy of the model was delineated at 88.24% (95% CI: 73.13% to 96.54%), highlighting the model's adept diagnostic proficiency in distinguishing between SS cases and non-SS cases.

## Discussion

The integration of AI, particularly through CNN as manifested by this autoML platform, heralds a new advancement in the diagnostic realm of SS and potentially streamlines the clinical or scientific workflow. The attained reliability score of 0.88 is emblematic of the profound potential that AI-driven methodologies hold in diagnostic pathology, aligning with the burgeoning narrative of AI's instrumental role in modern healthcare [15].

Despite a smaller dataset, the results of this autoML study are comparable to recently presented data from a conventionally programmed deep learning algorithm to detect FS [10]. The advantage of autoML platforms, however, is a more agile and faster approach that can also be used by biological scientists and clinicians to address more individual questions than the detection of FS, including preclinical analyses.

Histopathological evaluation, especially the assessment of FS, continues to be a linchpin in the diagnostic algorithm of SS. The objective quantification of FS via the autoML platform adeptly navigates the recognized challenge of interobserver variability, a notable impediment in SS diagnosis [16, 17]. This approach thereby fortifies the diagnostic criteria elucidated by the 2016 ACR/EULAR, accentuating the indispensable role of salivary gland biopsy in SS diagnosis [18, 19]. An alternative approach that could enhance the objectivity of our AI model is the quantification of lymphocytes per 4mm2, rather than relying solely on a binary classification based on Focus Score (FS). This method could provide more detailed insights into lymphocytic infiltration and potentially improve the correlation with clinical outcomes. Although this approach requires further development and validation, it represents a promising direction for future research. By using autoML Platforms, such as here for SS, the diagnostic process can theoretically be completely automated. In combination with less invasive biopsy methods of the salivary glands [20], the diagnosis of SS can thus be scaled and accelerated. The combined use of generative AI can also directly report the results of these algorithms and, after clinical validation, add them to the electronic medical record or create further predictive models in the context of clinical data.

The Giotto platform's utility extends to providing a nuanced understanding of the disease by allowing for the integration of histological, demographic, clinical, and immunological data. This not only aids in the diagnosis and classification of SS but also offers a comprehensive platform for investigating the systemic involvements and EGMs in SS patients and its association with histology.

The integration of AI in diagnostic workflows, such as the use of AutoAI/no-code solutions, faces several regulatory hurdles. For laboratory-developed tests, especially those involving AI algorithms, regulatory approval requires rigorous validation across diverse clinical settings. The democratization of AI tool development through no-code platforms can potentially accelerate this process by enabling pathologists and researchers to develop and test algorithms without extensive coding expertise. However, ensuring the robustness and generalizability of these algorithms across different populations and clinical conditions is crucial for gaining regulatory approval and widespread clinical adoption.

This study has several limitations. It emanates from the relatively modest cohort size and its single-center nature, which might potentially constrain the generalizability of the findings across diverse populations and clinical settings. Furthermore, the algorithm presented here was not evaluated in an external dataset, and an independent hold-out test set was not included due to the limited sample size, which may impact the generalizability of the model's performance. While the Giotto platform showcased notable accuracy in SS diagnosis, the transposition of AI algorithms to routine clinical practice necessitates a thorough understanding of the algorithm's performance across varied clinical and demographic landscapes. However, we would like to point out that autoML platforms such as Giotto provide user interfaces in the form of web apps that can be integrated in other software systems such as the electronic medical record. We postulate that autoML platforms do not serve to develop algorithms to be put into production and approved by regulatory bodies. On the other hand, they are so flexible that they have found their role in diagnostics, or at least preclinical research, in a different way. We do not show heatmaps in this study to demonstrate that the algorithm actually applied inflammatory foci to its decision. This could be added in future work as heatmaps have an educative value by drawing attention to diagnostically important structures by color [21].

## Conclusions

In conclusion, the encouraging findings from this study underscore the substantive promise of AI in enhancing diagnostic accuracy and efficiency in SS diagnosis. The systematic identification of complex histopathological features and their correlation with clinical manifestations harbors a promising avenue for future research endeavors [22]. AutoML platforms as presented here enable clinicians and researchers to develop their own algorithms, which pinpoints their large potential in horizontal and vertical digitalisation. Clearly, larger datasets with external evaluation are necessary to fully estimate the robustness of such algorithms.

## Data Availability

Datasets from this study are not publicly available due to privacy and ethical constraints but can be obtained from the corresponding author upon reasonable request.

## Abbreviations

SS: Sjögren's Syndrome
EGMs: Extraglandular Manifestations
ACR/EULAR: American College of Rheumatology/European League Against Rheumatism
FS: Focus Score
AI: Artificial Intelligence
FDA: Food and Drug Administration
CNN: Convolutional Neural Networks
autoML: Automated Machine Learning
H&E: Hematoxylin and Eosin
AUC: Area Under the Curve
CI: Confidence Interval
WUSF: Whole Unstimulated Salivary Flow
